# Aerobic Fitness Does Not Modify the Effect of *FTO* Variation on Body Composition Traits

**DOI:** 10.1371/journal.pone.0051635

**Published:** 2012-12-17

**Authors:** Antti Huuskonen, Jani Lappalainen, Niku Oksala, Matti Santtila, Keijo Häkkinen, Heikki Kyröläinen, Mustafa Atalay

**Affiliations:** 1 Institute of Biomedicine, Physiology, University of Eastern Finland, Kuopio, Finland; 2 Department of Surgery, Tampere University Hospital, Tampere, Finland; 3 Defence Command, Personnel Division, Finnish Defence Forces, Helsinki, Finland; 4 Department of Biology of Physical Activity, University of Jyväskylä, Jyväskylä, Finland; Universidad Europea de Madrid, Spain

## Abstract

**Purpose:**

Poor physical fitness and obesity are risk factors for all cause morbidity and mortality. We aimed to clarify whether common genetic variants of key energy intake determinants in leptin (*LEP*), leptin receptor (*LEPR*), and fat mass and obesity-associated (*FTO*) are associated with aerobic and neuromuscular performance, and whether aerobic fitness can alter the effect of these genotypes on body composition.

**Methods:**

846 healthy Finnish males of Caucasian origin were genotyped for *FTO* (rs8050136), *LEP* (rs7799039) and *LEPR* (rs8179183 and rs1137101) single nucleotide polymorphisms (SNPs), and studied for associations with maximal oxygen consumption, body fat percent, serum leptin levels, waist circumference and maximal force of leg extensor muscles.

**Results:**

Genotype AA of the *FTO* SNP rs8050136 associated with higher BMI and greater waist circumference compared to the genotype CC. In general linear model, no significant interaction for *FTO* genotype-relative VO_2_max (mL·kg^−1^·min^−1^) or *FTO* genotype-absolute VO_2_max (L·min^−1^) on BMI or waist circumference was found. Main effects of aerobic performance on body composition traits were significant (p<0.001). Logistic regression modelling found no significant interaction between aerobic fitness and *FTO* genotype. *LEP* SNP rs7799039, *LEPR* SNPs rs8179183 and rs1137101 did not associate with any of the measured variables, and no significant interactions of *LEP* or *LEPR* genotype with aerobic fitness were observed. In addition, none of the studied SNPs associated with aerobic or neuromuscular performance.

**Conclusions:**

Aerobic fitness may not modify the effect of *FTO* variation on body composition traits. However, relative aerobic capacity associates with lower BMI and waist circumference regardless of the *FTO* genotype. *FTO*, *LEP* and *LEPR* genotypes unlikely associate with physical performance.

## Introduction

Obesity and low physical fitness frequently associate with each other, and are individual risk factors for many pathological conditions and cardiovascular mortality [1], [2]. Body composition and physical performance are outcomes of cumulative effects of common and rare variants in a large number of genes with environmental and gene-gene interactions [3]. The effects of behavioural factors on human performance, including physical activity and dietary habits have been widely studied, yet knowledge on genetic background is sparse. The heritability estimates for obesity range from 40% to 70% [3], from 20 to 40% for aerobic performance [4], [5], and approximately 60% for muscle force [6]. Single nucleotide polymorphisms (SNPs) represent the most common type of genetic variation in the human genome [7]. However, it is not known whether genetic variants in genes encoding fat and obesity-associated (*FTO)*, leptin (*LEP),* and leptin receptor (*LEPR)* associate with physical performance, and whether fitness level modifies the risk for obesity associated with these gene variants.

Genome-wide association studies have identified the *FTO* gene as the first susceptibility locus for common obesity [8], [9]. Minor allele of this variant is associated with increased risk for obesity and elevated body weight [8], [9]. In a recent meta-analysis, physical activity level was shown to modify the relationship between the *FTO* risk variant and body weight and risk of obesity [10]. However, effects of *FTO* genotype and its interaction with aerobic fitness on body composition have not been reported so far. Despite the fact that *FTO* is under extensive research, the role and function of the *FTO* gene product remains incompletely understood.

Leptin is a peptide hormone secreted mainly from white adipose tissue. It regulates hunger, body temperature and energy metabolism, and has been used as a biomarker of energy deficiency [11], [12]. Recent studies have shown that obese individuals are in fact leptin resistant [12], and that physical exercise may restore leptin sensitivity [13]. Associations of leptin receptor gene (*LEPR)* SNP Gln223Arg (rs1137101) and leptin (*LEP*) promoter region SNP -2548G/A (rs7799039) with BMI and other body composition-related traits have been reported [14]–[18]. We previously demonstrated that the rs7799039 associates with changes in body composition in response to physical training [19]. Furthermore, another *LEPR* SNP Lys656Asn (rs8179183) has been reported to associate with substrate oxidation and basal metabolic rate [20], [21]. However, the interaction effects of these SNPs with aerobic performance on body composition are not known.

The present study investigated the association of selected SNPs in *FTO*, *LEP* and *LEPR* with body composition, neuromuscular and cardiorespiratory performance and plasma leptin levels in 846 healthy male subjects. In addition, genotype-aerobic fitness interactions on body composition traits were studied. Our hypothesis was that these common variants are associated with human performance, body composition and health-related risk factors. Furthermore, we hypothesized that aerobic fitness modifies the relationship between the genetic variants and body composition. The information this study provides may be used to identify those individuals having higher health risks and tendency for poor physical fitness, and for better understanding of the effect of genetic factors on human physical performance.

## Materials and Methods

### Study Subjects

The subjects of Caucasian origin were 846 healthy male Finnish volunteers with mean age (SD) of 25±5 years. All subjects were informed on the purpose of the study, gave a written informed consent and underwent medical examination prior to the tests. This study was approved by the ethics committee of the University of Jyväskylä and the hospital district of Central Finland. Anthropometric data and blood samples were collected after an overnight fast. The subjects consumed light breakfast 1 to 2 hours before the exercise tests.

### Physical Performance and Body Composition

Height, weight and waist circumference were recorded, and body mass index (BMI, kg·m^−2^) was calculated. Body fat and lean mass percentage were recorded by using the eight-polar bioimpedance method with multifrequency current (InBody 720; Biospace Company, Seoul, Korea). Bioimpedance was recorded after an overnight fast and with at least one day off from any intensive physical activity.

Maximal isometric force production of the leg extensor muscles was measured with a dynamometer (Department of Biology of Physical Activity, University of Jyväskylä, Finland). The test was performed in sitting position with 107-degree knee angle. The subjects were supervised to generate maximal force as fast as possible and maintain this force for 3 seconds. The data were analysed with a 16-bit AD converter (CED power 1401, Cambridge Electronic Design ltd, England) and Signal (2.16) software.

Aerobic fitness was assessed by maximal bicycle ergometer test (Ergoline 800 S, Ergoselect 100 K, Ergoselect 200 K, Bitz, Germany) as previously described [22]. The initial workload was 50 W with 25 W increase on 2-minute intervals until exhaustion. Heart rate was monitored throughout the test (Polar T-31; Polar Vantage, Kempele, Finland). The analyzed variables were maximum heart rate, maximal workload and maximal oxygen consumption (mL·kg^−1^·min^−1^) estimated by software (Milfit4/Fitware, Finland) [(11.016 · maximum work load) x (1·body weight^−1^) +7.0]. The test was terminated when the subject could not maintain the required cycling speed (60–90 rpm). Physical activity was assessed with international physical activity questionnaire (IPAQ) [23].

### Blood Samples and Genotyping

The fasting blood samples were collected and analysed immediately with a hemacytometer (Sysmex Co., Kobe, Japan). Plasma was separated from the whole blood and stored at -80C° until analysis. Plasma leptin concentrations were assayed by commercial ELISA according to the manufacturer’s instructions (Quantikine, R&D Systems, Minneapolis, MN, USA). Assay specifications were as follows: sensitivity limit 7.8 pg·mL^−1^, and maximum intra- and interassay CV% 3.3% and 5.4%, respectively.

The SNP genotyping was performed using allele-specific PCR assays. Briefly, genomic DNA was first isolated from the blood mononuclear cells using QIAamp DNA Blood kit (Qiagen, Hilden, Germany). Next, 50 nanograms of the DNA was amplified with Brilliant QPCR Master Mix (Stratagene, La Jolla, CA, USA) and allele-specific SNP assays on a Mx3000P Real-time PCR System (Stratagene). For *FTO* (rs8050136) and *LEPR* SNP Lys656Asn (rs8179183), the commercially available TaqMan SNP genotyping assays were used (Applied Biosystems, Foster City, CA, USA), and for *LEP* (rs7799039) and *LEPR* SNP Gln223Arg (rs1137101), the following molecular beacons SNP genotyping assays were used: LEP SNP forward primer 5′-CCTGTAATTTTCCCATGAGAAC-3′ and reverse primer 5′-TGCAACATCTCAGCACTTAG-3′, and the molecular beacons 5′-FAM/HEX-CGTGCCCGACAGGGTTGC(G/A)CTGATCGGCACG -BHQ1; LEPR SNP forward primer 5′-TCAACGACACTCTCCTTATG-3′ and reverse primer 5′-TTATGGGCTGAACTGACAT-3′, and the molecular beacons 5′-FAM/HEX- CGGACGTGGAGTAATTTTCC(A/G)GTCACCTCCGTCCG -BHQ1-3′.

### Statistics

Calculations were performed with SPSS software (Illinois, Chicago, USA) by using one-way ANCOVA or non-parametric tests, when appropriate. Age, smoking and earlier physical activity were set as covariates. Smoking (smoker or non-smoker) and physical activity (vigorous physical activity more than 3 times per week) were set as dichotomous variables, whereas age VO_2_max, BMI, waist circumference were set as continuous variables.

Genotype-VO_2_max interaction on BMI, waist circumference and fat percent were analyzed by using general linear model. The general linear model included main effects terms for earlier physical activity, smoking, age, genotype, VO_2_max and genotype-VO_2_max interaction. Physical activity and smoking were set as dichotomous variables, and VO_2_max, age and body composition traits were set as continuous variables. Genotype was set as discrete variable in every analysis.

Additionally, the *FTO* genotype and *FTO* genotype-VO_2_max interaction related odds for overweight (BMI over 25 kg·m^−2^) and abdominal obesity (waist circumference over 90 cm) were analyzed by logistic regression. Recessive model was applied in analysis (AA genotype vs. CA and CC genotype). Age, smoking and physical activity were set as covariates. The subjects were divided into quartiles according to VO_2_max. The 75% percentile limits for VO_2_max were 3.7 L·min^−1^ and 46.9 mL·kg^−1^·min^−1^. The subjects in the highest 25% quartile were defined as high aerobic fitness group, and compared with the 75% percentile of subjects (VO_2_max <3.7 L·min^−1^ or <46.9 mL·kg^−1^·min^−1^).

Data are presented as mean ± standard deviation unless otherwise stated. Statistical significance was set at p<0.0125 to account for multiple testing. Bonferroni correction was applied to post-hoc comparisons. Statistical power and sample sizes were estimated with SISA web calculator [24]. A 90% level was chosen for power calculations, and the number of subjects needed to demonstrate 1 S.D. difference in continuous parameters was estimated.

## Results

All SNPs conformed to Hardy-Weinberg’s equilibrium. None of the SNPs were associated with earlier physical activity estimated with IPAQ.

**Figure 1:**
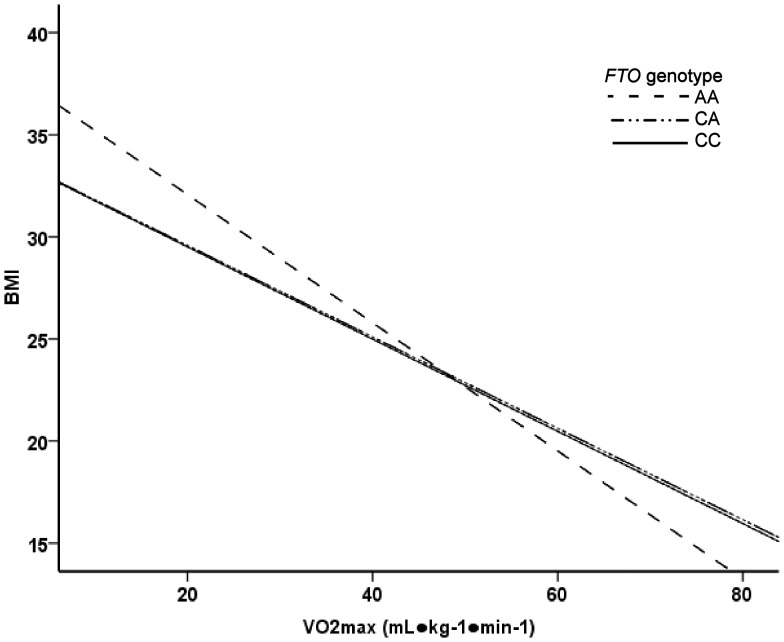
Linear regression analysis. The main effect of VO_2_max (mL·kg^−1^·min^−1^) on BMI was significant (p<0.001). The main effects of *FTO* genotype and *FTO* genotype-VO_2_max (mL·kg^−1^·min^−1^) interaction were not significant (p = 0.029 and p = 0.081, respectively).

**Figure 2:**
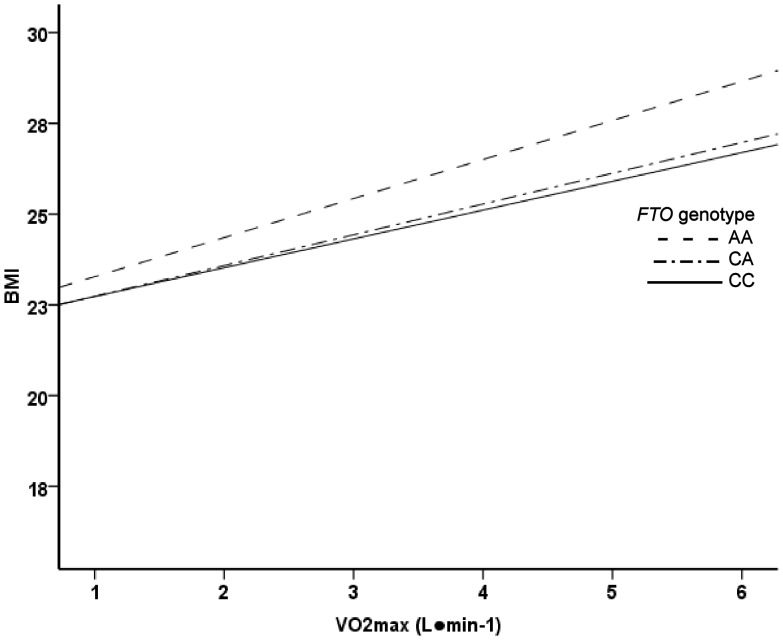
Linear regression analysis. The main effect of VO_2_max (L·min^−1^) on BMI was significant (p<0.001). The main effects of *FTO* genotype and *FTO* genotype-VO_2_max (L·min^−1^) interaction were not significant (p = 0.964 and p = 0.937, respectively).

**Figure 3:**
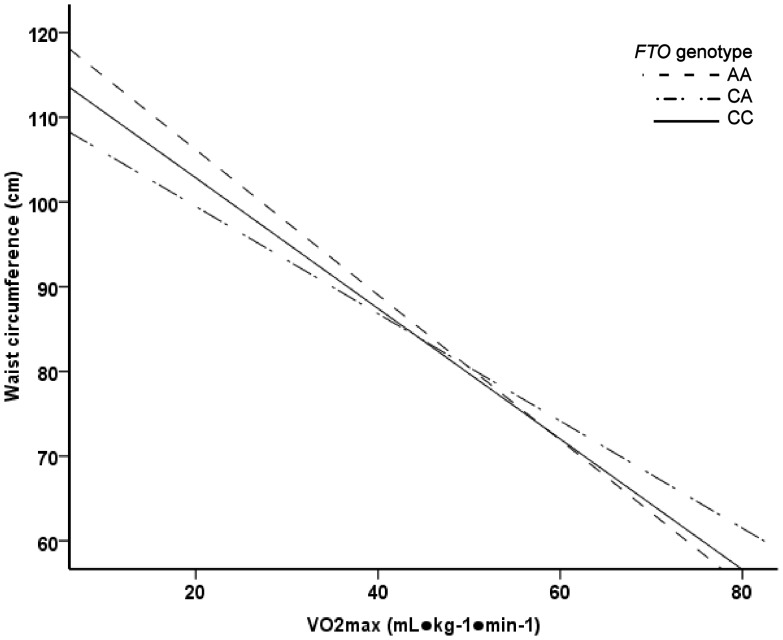
Linear regression analysis. The main effect of VO_2_max (mL·kg^−1^·min^−1^) on waist circumference was significant (p<0.001). The main effects of *FTO* genotype and *FTO* genotype-VO_2_max (mL·kg^−1^·min^−1^) interaction were not significant (p = 0.040 and p = 0.093, respectively).

**Figure 4:**
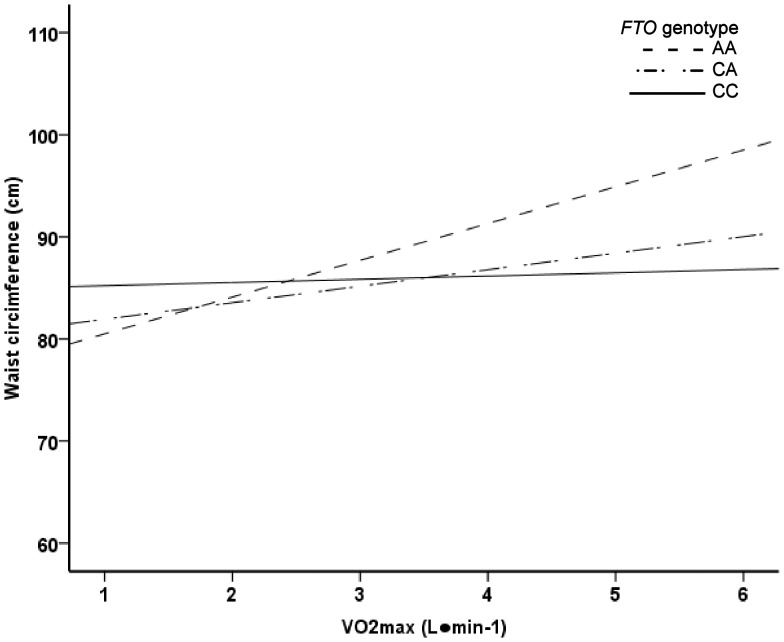
Linear regression analysis. The main effect of VO_2_max (L·min^−1^) on waist circumference was significant (p<0.001). The main effects of *FTO* genotype and *FTO* genotype-VO_2_max (L·min^−1^) interaction were not significant (p = 0.521 and p = 0.262, respectively).

In ANCOVA analysis, the *FTO* SNP rs8050136 associated with BMI and waist circumference (Table 1). Genotype AA carriers had significantly higher BMI and greater waist circumference compared to the genotype CC carriers. In general linear model, the main effects of relative and absolute VO_2_max on BMI and waist circumference were significant (p<0.001). However, no significant *FTO* genotype-relative VO_2_max (mL·kg^−1^·min^−1^) interaction on BMI (p = 0.081) or waist circumference (p = 0.093) was found. In addition, the test result was insignificant when *FTO* genotype-absolute VO_2_max (L·min^−1^) interaction on BMI (p = 0.937) or waist circumference (p = 0.262) were analyzed (Figures 1–4). In logistic regression, genotype AA increased odds for waist circumference over 90 cm. However, the interaction of genotype AA with high VO_2_max was insignificant (Table 2). No association was found with aerobic fitness (VO_2_max expressed as L·min^−1^ or mL·kg^−1^·min^−1^) (Table 1).

**Table 1:** Showing the results of ANCOVA–analysis according to *FTO* genotype.

	*FTO* SNP rs8050136 (N = 773)		
Genotype	CC	CA	AA
	N = 269	N = 380	N = 124
	35%	49%	16%
**BMI (kg· m** ^−**2**^ **)**	24.5±3.6	24.8±3.7	25.7±4.3*
**Waist circumference (cm)**	85.8±10.3	86.0±9.7	88.8±12.2#
**Fat per cent (%)**	17.5±7.2	17.9±7.0	19.4±8.0
**VO2max (mL·kg** ^−**1**^ **·min** ^−**1**^ **)**	42.0±8.3	41.8±7.8	40.2±8.6
**VO2max (L·min** ^−**1**^ **)**	3.29±0.56	3.30±0.58	3.30±0.62
**Maximal working capacity (Watts)**	244.2±44.7	245.0±46.5	242.7±49.1
**Maximal force of leg** **extensors (N)**	2927±872	2950±816	2942±992
**Plasma leptin (pg·mL** ^−1^ **)**	3598±3657	3702±3780	4289±4278

Earlier physical activity, smoking and age were set as covariates. Bonferroni correction was accounted for multiple post-hoc comparisons.

BMI: p = 0.007 for main effect, *p = 0.005 between the genotype groups CC and AA.

Waist circumference: p = 0.012 for main effect, #p = 0.012 between the genotype groups CC and AA.

p>0.0125 for main effect between genotype groups for rest of the variables.

**Table 2:** Showing results of the logistic regression analysis.

	OR	95% C.I.	p-value
**BMI over 25 kg·m** ^−**2**^ ** adjusted for age, smoking, physical activity and relative aerobic fitness (mL·kg** ^−**1**^ **·min** ^−**1**^ **)**			
Genotype AA	1.51	0.96–2.37	0.073
VO_2_max (>46.9 mL·kg^−1^·min^−1^)	0.15	0.09–0.25	<0.001
Genotype AA by VO_2_max (>46.9 mL·kg^−1^·min^−1^)	1.31	0.43–3.96	0.639
**BMI over 25 kg·m** ^−**2**^ ** adjusted for age, smoking, physical activity and absolute aerobic fitness (L·min** ^−**1**^ **)**			
Genotype AA	1.61	1.02–2.53	0.038
VO_2_max (>3.7 L·min^−1^)	1.10	0.75–1.63	0.626
Genotype AA by VO_2_max (>3.7 L·min^−1^)	1.02	0.41–2.58	0.959
**Waist circumference over 90 cm adjusted for age, smoking, physical activity and relative aerobic fitness (mL·kg** ^−**1**^ **·min** ^−**1**^ **)**			
Genotype AA	1.92	1.22–3.03	0.005
VO_2_max (>46.9 mL·kg^−1^·min^−1^)	0.08	0.04–0.18	<0.001
Genotype AA by VO_2_max (>46.9 mL·kg^−1^·min^−1^)	1.07	0.19–5.94	0.941
**Waist circumference over 90 cm adjusted for age, smoking, physical activity and absolute aerobic fitness (L·min** ^−**1**^ **)**			
Genotype AA	1.96	1.22–3.15	0.005
VO_2_max (>3.7 L·min^−1^)	1.26	0.81–1.97	0.299
Genotype AA by VO_2_max (>3.7 L·min^−1^)	0.88	0.33–2.40	0.884

OR and 95% C.I. for the genotype AA (vs. genotype CA and CC) of the *FTO* rs8050136 according to overweight and abdominal obesity.

In ANCOVA analysis, *LEP* SNP rs7799039 did not associate with any variable (Table 3). No interactions were observed between aerobic fitness and genotype. *LEPR* SNP rs1137101 did not associate with BMI, body fat, leptin levels or physical performance in ANCOVA (Table 3), and no aerobic performance-genotype interactions were observed in the general linear model. In ANCOVA analysis, *LEPR* Lys656Asn SNP rs8179183 did not associate with any variable (Table 3). In addition, no interactions were observed between genotype and aerobic fitness in the general linear model.

**Table 3:** Showing the results of ANCOVA –analysis according to *LEP* and *LEPR* genotypes.

		*LEP* -2548 SNP rs7799039 (N = 713)			*LEPR* Lys656Asn SNP rs8179183 (N = 713)			*LEPR* Gln223Arg SNP rs1137101 (N = 713)		
Genotype		GG	GA	AA	Lys/Lys	Lys/Asn	Asn/Asn	AA	AG	GG
		N = 176	N = 384	N = 153	N = 549	N = 152	N = 12	N = 125	N = 350	N = 238
		25%	54%	21%	77%	21%	2%	18%	49%	33%
**BMI (kg·m** ^−**2**^ **)**		25.0±3.8	24.9±3.9	24.4±3.4	25.0±3.8	24.4±3.8	23.6±2.1	24.5±3.4	24.9±3.9	24.9±3.8
**Waist circumference (cm)**		86.6±10.1	86.6±10.8	85.4±9.6	86.8±10.6	84.6±9.8	83.9±4.9	86.2±9.8	86.3±10.3	86.5±10.9
**Fat per cent %**		18.5±7.2	18.1±7.4	17.1±7.0	18.2±7.3	17.2±7.4	16.8±6.0	18.1±6.9	17.6±7.5	18.4±7.1
**VO_2_max (mL·kg** ^−**1**^ **·min** ^−**1**^ **)**		40.7±8.3	42.1±8.2	41.7±7.7	41.3±8.1	42.8±8.2	40.2±6.1	42.5±8.3	41.7±8.1	41.1±8.0
**VO_2_max (L·min** ^−**1**^ **)**		3.26±0.55	3.33±0.60	3.27±0.60	3.29±0.58	3.33±0.60	3.09±0.42	3.34±0.58	3.31±0.58	3.27±0.58
**Maximal working capacity (Watts)**		240.4±44.6	246.9±47.3	242.5±45.4	243.8±45.7	247.9±48.1	228.4±33.9	248.1±46.1	245.0±46.6	241.5±45.9
**Maximal force of leg extensors (N)**		2952±835	2942±863	2920±901	2948±837	2937±967	2586±630	2913±758	2958±847	2928±937
**Plasma leptin (pg·mL** ^−**1**^ **)**		3738±3686	3858±3961	3517±3635	3746±3679	3842±4 398	3069±2827	4161±4378	3781±4057	3513±3102

Earlier physical activity, smoking and age were set as covariates. Bonferroni correction was accounted for multiple post-hoc comparisons.

p>0.0125 for main effect between genotype groups for every SNP.

## Discussion

The main finding of the present study was that aerobic fitness does not modify the effect of *FTO* variation on BMI or waist circumference. However, relative aerobic capacity did associate with lower BMI and waist circumference regardless of the *FTO* genotype. We also report that the *LEP* promoter -2548 SNP and the *LEPR* Lys656Asn SNP did not associate with BMI, waist circumference or body fat percent.

Our observations that genotype AA of the *FTO* variant associated with higher BMI and greater waist circumference are in line with a recent meta-analysis [25]. Our novel finding, however, is that aerobic fitness does not modify the effect of *FTO* variation on BMI and waist circumference. In another recent meta-analysis, the level of physical activity was shown to modify the relationship between the *FTO* risk variant and body weight and risk of obesity [10]. Nevertheless, these studies are not fully comparable to ours because at present, this is the first study to report objectively measured aerobic fitness - *FTO*-interaction. The questionnaire-based assessment of physical activity may have some reporting bias [26]. In addition, the present findings may be explained by the fact that apart from physical activity, other factors may affect aerobic fitness. Approximately 20 to 40% of VO_2_max has been estimated to be heritable, and other factors, such as physical activity, intake of carbohydrates, smoking, body weight, blood haemoglobin levels, age and presence of chronic disease account for the remaining [4], [5]. In addition, the present and earlier studies differ in study design because in our study, all subjects were young males. Nevertheless, in the present study, aerobic fitness was associated with body composition traits regardless of the *FTO* genotype. Despite lack of *FTO* genotype-aerobic fitness interaction, the genotype AA carriers are more susceptible to obesity and may benefit from increased aerobic fitness to a greater extent due to its favourable effects on the regulation of body weight. In the present study, *FTO* variant was not found to associate with aerobic fitness, which is in line with previous studies [27], [28]. It can be speculated that low physical fitness combined with increased body weight in the allele A carriers of the *FTO* variant may further increase risk for lifestyle-associated disease, such as cardiovascular events [29]. This would in turn highlight the importance of physical activity to attenuate risk for obesity. Furthermore, the studied *FTO* variant did not associate with plasma leptin levels, which in line with other studies [30], [31].

In the present study, the *leptin* promoter SNP rs7799039 did not associate with BMI, which is supported by earlier reports [17], [18]. It has been speculated that lower leptin levels in allele G carriers of this SNP would be an underlying mechanism for increased accumulation of adipose tissue [32]. However, in the present study, leptin levels were similar between the genotype groups. Furthermore, no association with aerobic fitness, neuromuscular performance, or interaction with VO_2_max was found.

The *LEPR* Gln223Arg genotype showed no association with cardio-respiratory fitness, which is supported by our previous report [19]. Furthermore, in agreement with other studies [19], [33], no differences in leptin levels between the genotype groups were found, although controversial findings does exist [15], [34], [35]. It should be noted, however, that in many published studies, interpretation of the results is limited due to small sample size, differences in ethnicity of the subjects, and other factors related to the study settings.

The *LEPR* Lys656Asn SNP did not associate with BMI, waist circumference or body fat per cent, and these findings are also supported by others [36]–[38]. Moreover, subjects of the present study were not morbidly obese, and small prevalence of the minor Asn allele may also complicate evaluation of the effect of this SNP on body weight and body composition. Furthermore, no significant genotype-VO_2_max interactions were found.

There are certain limitations to the present study. First, the sample size was moderate for genetic association analyses. The relatively large confidence intervals may bias identification of a true interaction in logistic regression analysis. In addition, all subjects were males, which may affect generalization of the results to other populations. Our study design was cross-sectional, which may increase risk for selection bias. However, earlier physical activity status was assessed with IPAQ survey, and no associations with pre-study physical activity and genotypes were found. The bioimpedance method is both sensitive and specific for analysis of body fat mass, but may have limitations for accurate evaluation of muscle mass [39]. The method for assessing aerobic fitness was indirect, whereas direct gas exchange analysis would provide greater accuracy.

In conclusion, aerobic fitness may not modify the effect of *FTO* variation on body composition traits. However, relative aerobic capacity had a favourable effect on BMI and waist circumference, regardless of the *FTO* genotype. It is unlikely that *FTO*, *LEP* and *LEPR* genotypes associate with physical performance. These findings can be applied in sports medicine to decrease risk for diseases related to sedentary lifestyle and obesity, where improved aerobic fitness is beneficial regardless of the *FTO-*related predisposition to obesity.
